# Case Report: Suspected Solitary Osseous Plasmacytoma in a Cat: Use of Magnetic Resonance Imaging to Diagnose and Confirm Resolution of Disease Following Chemotherapy

**DOI:** 10.3389/fvets.2021.752279

**Published:** 2021-10-05

**Authors:** Talisha M. Moore, Stephanie A. Thomovsky, Craig A. Thompson, Hock Gan Heng, R. Timothy Bentley

**Affiliations:** ^1^Department of Veterinary Clinical Science, Purdue University, West Lafayette, IN, United States; ^2^Department of Comparative Pathobiology, Purdue University, West Lafayette, IN, United States

**Keywords:** magnetic resonace imaging, vertebral tumor, hyperglobulinemia, monoclonal gammaopathy, feline

## Abstract

A 9-year-old female spayed Domestic Shorthair cat presented for pain, reluctance to jump, and hyporexia of 14 days duration. Neurologic examination was consistent with C6-T2 myelopathy. Magnetic resonance imaging (MRI) revealed a solitary, contrast-enhancing lesion within the T2 vertebral body. Solitary osseous plasmacytoma was diagnosed based on neurologic examination, advanced imaging, and clinicopathologic findings. Melphalan and prednisolone therapy were initiated. Complete resolution of clinical signs and the vertebral lesion were documented at a 2-year follow up examination with neurologic examination and repeat spinal MRI, respectively. Solitary osseous plasmacytoma are rare neoplasms in humans and domestic animals. As such, there is a paucity of published information regarding diagnostic criteria, MRI findings, treatment modalities, progression, and remission of disease in the feline patient. Most data are extrapolated from human medicine. The purpose of this report is to document neurologic exam and MR findings at the time of diagnosis and complete resolution of a solitary osseous vertebral plasmacytoma following melphalan and prednisolone therapy.

## Introduction

Myeloid related disorders (MRD) are resultant of malignant propagation of immunoglobulin-secreting B lymphocyte precursors or plasma cells and manifest as disseminated disease or a solitary plasmacytoma. MRD can be further classified as; multiple myeloma (MM), cutaneous extramedullary plasmacytoma, non-cutaneous extramedullary plasmacytoma, solitary osseous plasmacytoma (SOP), IgM macroglobulinemia, immunoglobulin-secreting lymphoma, immunoglobulin-secreting leukemia, and plasma cell leukemia. Across species, plasma cell neoplasms are relatively rare. These tumors represent <0.1% of all feline neoplasms (1, 2) and ≤ 1% of all canine neoplasms (2, 3). In humans the there is a cumulative incidence of 0.15/100.00 (4).

Plasma cell neoplasms may have myriad manifestations but clinical presentation in veterinary medicine tends to be non-specific with lethargy, anorexia, and weight loss most reported (2, 5–8). However, when these tumors affect vertebra, common clinical signs are pain and neurologic deficits consistent with neuroanatomic localization (9–11). Solitary plasmacytoma involves a single lesion of neoplastic plasma cells and no or insignificant bone marrow plasmacytosis. They may present as an extramedullary lesion, which can be further divided into cutaneous or non-cutaneous lesions without osseous involvement, or as a solitary osseous lesion (2). Osseous plasmacytoma tend to have a predilection for marrow-containing bones like vertebrae, the pelvis or femurs.

Currently, there are no published diagnostic criteria for definitive diagnosis of SOP in the feline patient. Extrapolating from human medicine, diagnosis necessitates advanced imaging confirming a single lesion, histopathological confirmed plasmacytoma, bone marrow biopsy confirming no, or insignificant bone marrow plasmacytosis, and immunohistochemical findings consistent with SOP. Solitary plasmacytomas are considered a precursor to multiple myeloma, a more malignant manifestation of a myeloid related neoplasia, but much of this information is extrapolated from human medicine (4, 12–14) and scarce case reports in the veterinary literature (15, 16). Because of the rarity of this neoplasm in cats, there is a paucity of published veterinary literature regarding the diagnostic criteria, gold-standard treatment, efficacy of treatment modalities, disease progression, and prognosis. To the authors' knowledge, there is a single published case report (9) describing the successful treatment of two feline patients with solitary plasmacytoma of bone with radiotherapy and chemotherapy, but advanced imaging was not used to diagnose or confirm resolution in these cases. This is the first case report describing the use of MRI to diagnose and document complete resolution of this neoplasm in the feline patient following treatment with solely chemotherapy.

## Case Presentation

A 9-year-old female spayed indoor-only American Domestic Shorthair cat (3.72 kg, 8.20 lb) was presented for progressive ill-defined pain, malaise, in appetence, and reluctance to jump. Fourteen days prior, the primary veterinarian had evaluated the cat. At that time, physical and neurologic examinations were unremarkable. Diagnostics performed included a complete blood count (Protocyte Dx, Idexx Laboratories, Westbrook, ME, USA), serum chemistry panel (Catalyst Dx, Idexx Laboratories, Westbrook, ME, USA), and thyroid level (SNAPshot Dx, Idexx Laboratories, Westbrook, ME, USA. Abnormalities identified on the CBC included leukopenia (2.83 K/μL, reference interval 2.87–17.02 K/μl), eosinopenia (0.09 K/μl, reference interval 0.17–1.57 K/μl), and thrombocytopenia (120 K/μl, reference interval 151–600 K/μl). The serum panel was suggestive of an inflammatory process with hyperproteinemia (9.2 g/dl, reference interval 5.7–8.9 g/dl) characterized by hyperglobulinemia (6.0 g/dl, reference interval 2.8–5.1 g/dl). The total thyroxine level was within the normal range. The patient was prescribed buprenorphine (0.04 mg/kg PO q 8–12 h) for pain with recommendations for activity restriction and monitoring at home. Clinical signs did not resolve, and cervical, thoracic, and abdominal radiographs were performed which were within normal limits. A presumptive diagnosis of soft tissue injury was made and recommendations given for continued activity restriction and pain management with buprenorphine. Due to refractory pain, the patient was presented to the emergency service at our institution 11 days later.

On presentation, abnormalities noted on physical examination included pain on palpation of the caudal aspect of the humeri. Abnormal neurologic findings were anisocoria, left pupil miosis, bilaterally decreased flexor withdrawal reflex of thoracic limbs and pain upon palpation of the tricep muscles bilaterally. These findings were consistent with a neuroanatomic localization of C6-T2 spinal cord segments, left lateralized. Differential diagnoses were neoplasia and meningomyelitis. Four days later the patient presented for further work-up including repeat blood work, MR imaging and cerebrospinal fluid (CSF) analysis.

Complete blood count abnormalities were leukopenia (3.8 K/μl, reference range: 6.0–18.0 K/μl), characterized by neutropenia (2.6 K/μl, reference range: 3.0–12.0 K/μl) and lymphopenia (0.9 K/μl, reference range: 1.5–7.0 K/μl), and thrombocytopenia (191 K/μl, reference range: 3.0–12.0 K/μl). Serum panel abnormalities included hyperproteinemia (9.7 g/dl reference range: 5.5–7.1 g/dl) characterized by hyperglobulinemia (6.1 g/dl, reference range: 2.3–3.8 g/dl) and bilirubinemia (0.6 mg/dl reference range: 0.0–0.4 mg/dl). Serological immunoassays for feline leukemia virus (FeLv) antigen and feline immunodeficiency virus (FIV) antibody test (SNAP^*^ Combo, Idexx Laboratories, Toronto, ON, Canada) were both negative. Urinalysis obtained via cystocentesis indicated normal renal concentrating ability (urine specific gravity, 1.053) and 1+ protein with negative blood contamination.

**Figure 1:**
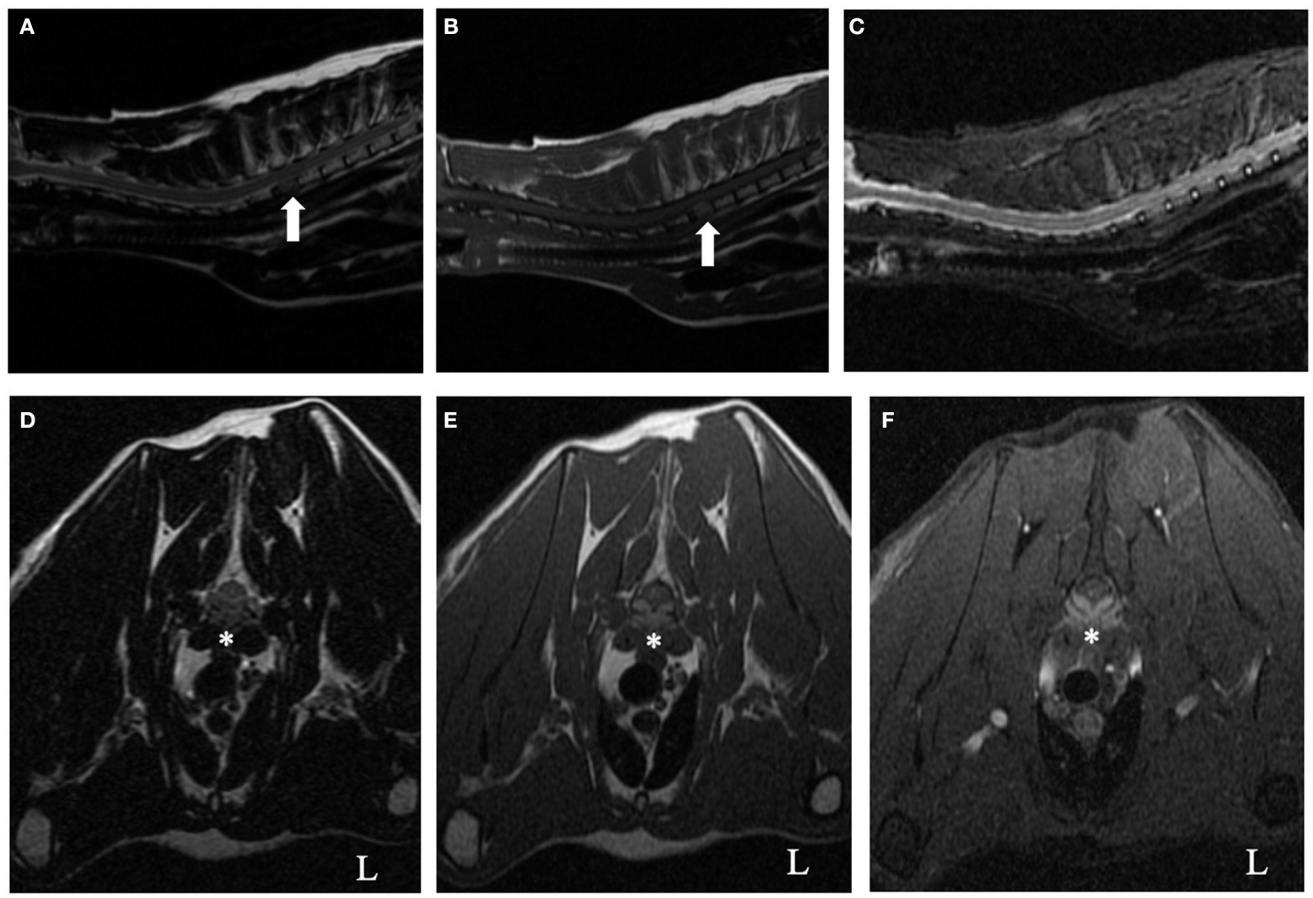
T2-weighted **(A,D)**, T1-weighted **(B,E)**, short tau inversion recovery (STIR) **(C)**, and T1-weighted contrast fat saturation **(F)** cervicothoracic images of a 9-year-old cat. On the sagittal T2-weighted and T1-weighted images (**A** and **B**, respectively), notice the homogenous reduction of signal intensity of the T2 vertebral body (arrow) relative to all other vertebrae. There was no abnormal hyperintense signal observed within the vertebral body on the STIR sagittal image **(C)**. At the level of mid-body of the T2 vertebra, there was a symmetric, ventrally located, extradural lesion causing secondary spinal cord compression (asterisk, **D–F**). The lesion was isointense relative to spinal cord parenchyma on T2-weighted **(D)** and hyperintense on T1-weighted non-contrast images and T1-weighted contrast and fat saturation images (**E,F**, respectively).

Cervicothoracic spine MRI with a 1.5-Telsa unit (GE Signa LXi, Waukesha, WI) revealed a diffuse homogeneous reduction of T1-weighted and T2-weighted signal intensity of the second thoracic vertebral body (T2) in comparison to normal bone (Figures 1A,B). There was no abnormal hyperintense signal observed within the vertebral body on short tau inversion recovery (STIR) (Figure 1C). The caudal aspect of the T2 vertebra was T1-hyperintense post gadopentetate administration. At the level of mid-body of the T2 vertebra, there was a symmetric, extradural lesion causing ventral spinal cord compression. The lesion was isointense relative to spinal cord parenchyma on T2-weighted and hyperintense on T1-weighted non-contrast images and T1-weighted contrast and fat saturation images (Figures 1D–F). Lumbar cerebrospinal fluid had an elevated protein concentration (73.4 mg/dl, reference interval <45 mg/dl) and total nucleated cell count (8 TNCC/μl, reference interval <5 TNCC/μl). Cytologic evaluation revealed no evidence of neoplasia or infectious agents. The considerably increased protein was suggestive of an inflammatory process within the central nervous system or compromise of the blood brain barrier. Considering the persistent hyperglobulinemia in conjunction with the presence of a solitary, contrast-enhancing vertebral body lesion on MRI, a round cell neoplasia or infectious granuloma became the most likely etiological differentials. Submission of infectious disease titers (Toxoplasma gondii, Cryptococcus spp.), protein electrophoresis, abdominal ultrasound, bone marrow aspirate and core biopsy were recommended to the owner. Sampling from the T2 vertebral body was not performed because of its challenging location to access without surgical intervention.

Abdominal ultrasound revealed small, poorly defined hypoechoic and hyperechoic nodules within the spleen. The remainder of the abdominal ultrasound was normal. Ultrasound-guided fine needle aspirates of the liver and spleen were performed; cytologic evaluation of the liver was unremarkable and extramedullary hematopoiesis was reported from the splenic nodules. Bone marrow (right humerus) aspirate revealed myeloid, erythroid, and megakaryocytic hypoplasia. Plasma cells along with small lymphocytes and activated macrophages were noted but comprised <1% of the total nucleated cell population. All nucleated cells had unremarkable morphology. There was no evidence of infectious agents, inflammation, or neoplastic cells. Following decalcification, the biopsy core sample contained only normal cortical bone—the sample was devoid of marrow. To further assess the persistent hyperglobulinemia, serum samples were submitted (Idexx Laboratories) for the purpose of protein electrophoresis, Cryptococcus antigen and Toxoplasma antibody titers. A distinct peak in the gamma range (Gamma 1, 3.31 g/dl, reference range: 0.3–2.5 g/dl) was identified with no increase in any other proteins, confirming monoclonal gammopathy. Urine was negative for Bence-Jones proteins (Idexx Laboratories). Cryptococcus antigen titer was negative. Toxoplasma IgG indirect immunofluorescence assay (IFA) titer was positive (1:1,600), IgM was negative, suggestive of exposure rather than active infection. Recommendations were made to repeat convalescent Toxoplasma titers in 2–3 weeks. Given all the diagnostic results, a presumptive diagnosis of solitary plasma cell tumor of the T2 vertebral body with secondary spinal cord compression was made. The patient was discharged on buprenorphine (0.04 mg/kg PO q 8–12 h) and gabapentin (6.7 mg/kg PO q 8 h) for pain control and referred to oncology for consultation.

One month later, the patient was presented to the oncology service for consultation and repeat diagnostics to obtain a more definitive diagnose. Neurologic consultation revealed progressive clinical signs of ambulatory tetraparesis with mild proprioceptive ataxia. The patient now exhibited a “two-engine” gait and proprioceptive placing deficits in both pelvic limbs with the left worse than right. Previously noted anisocoria and bilaterally decreased flexor withdrawal reflex of thoracic limbs were static. Repeat CBC and chemistry panel, abdominal ultrasound with FNA of the liver and spleen, and submission of convalescent Toxoplasma titers was performed. Repeat bone marrow core biopsy was declined due to concerns of morbidity. Complete blood count abnormalities were persistent leukopenia (4.2 K/μl, reference range: 6.0–18.0 K/μl) and thrombocytopenia (218 K/μl, reference range: 3.0–12.0 K/μl). Serum panel abnormalities included persistent hyperglobulinemia (5.5 g/dl, reference range: 2.3–3.8 g/dl). Repeat ultrasound revealed no significant findings. Fine needle aspirates of the liver and spleen were repeated and revealed minimal vacuolar hepatopathy with mild plasma cell infiltration and minimal lymphoid reactivity, respectively. While convalescent toxoplasmosis titers were pending, the patient was discharged with empirical prednisolone (1.5 mg/kg PO q 24 h) and clindamycin (13 mg/kg PO q 12 h) therapy for the presumptive treatment of plasma cell tumor and toxoplasmosis. Over the following 3 days, neurologic status improved. Clients were offered but declined radiation therapy for the treatment of the T2 vertebral lesion. The convalescent Toxoplasma titer was unchanged, confirming exposure rather than active infection. Clindamycin therapy was discontinued, prednisolone was tapered (1 mg/kg PO q 24 h) and empirical therapy with melphalan (0.1 mg/kg PO q 24 h) was initiated. The patient did well, eventually being tapered to a lower dose of melphalan (0.1 mg/kg PO q 72 h) and prednisolone (0.5 mg/kg PO q 24 h) at which she was maintained.

**Figure 2:**
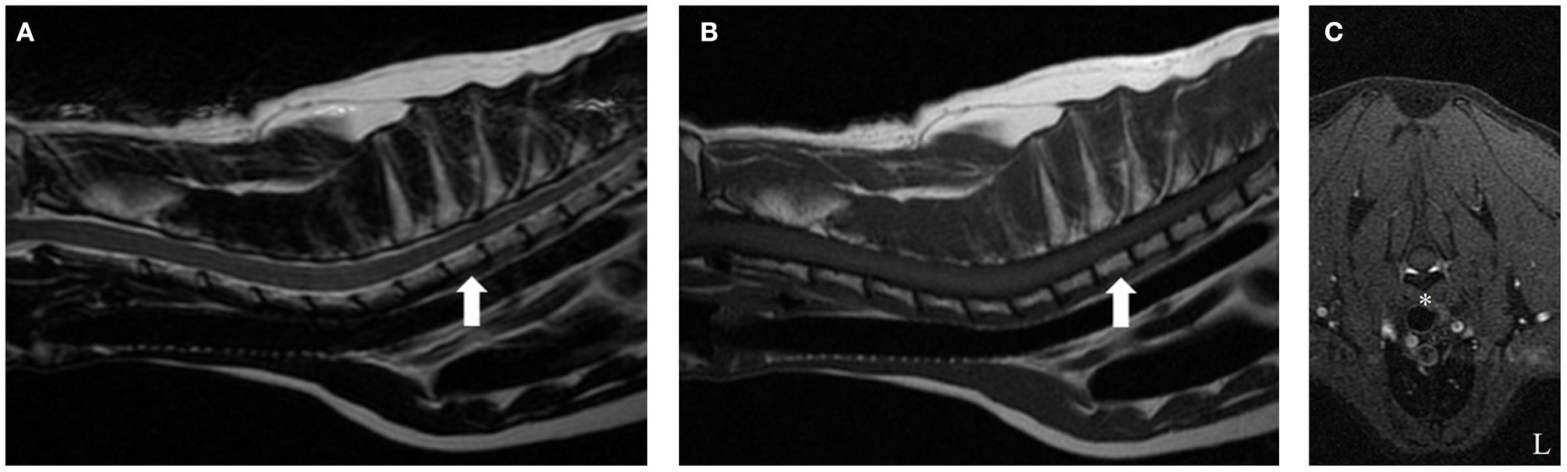
T2-weighted **(A)**, T1-weighted **(B)**, and T1-weighted fat suppression post-contrast **(C)** cervicothoracic images of the same cat 2 years after starting melphalan. On the sagittal T2-weighted and T1-weighted images (**A,B**, respectively), notice the isointense signal intensity of the T2 vertebral body (arrow) relative to all other vertebrae. The previously noted symmetric, ventrally located, extradural lesion causing secondary spinal cord compression at the level of mid-body of the T2 vertebra has resolved. There is no contrast enhancement on T1-weighted contrast fat saturation images (asterisk, E).

**Figure 3:**
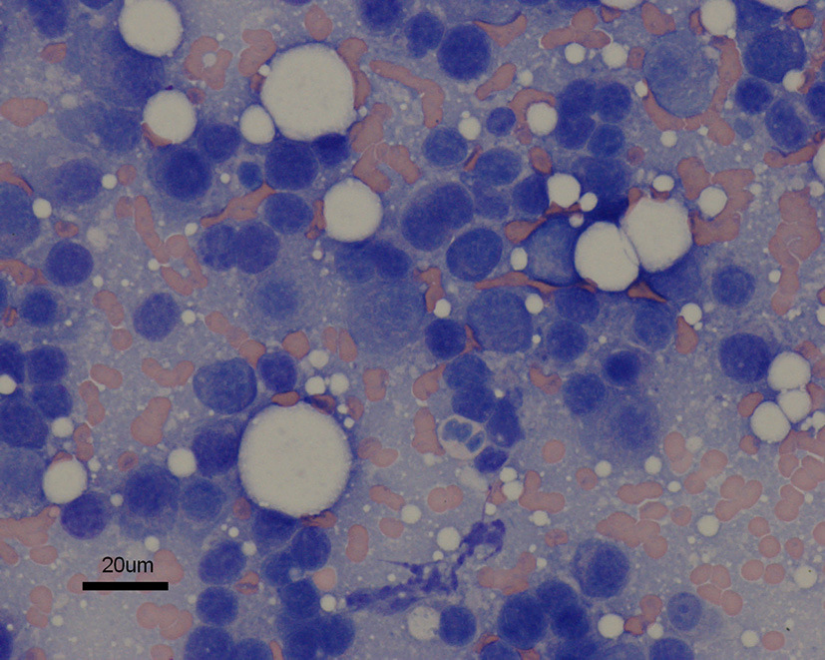
Fine needle aspirate of cutaneous tail base mass. The discrete cells are small to medium in size and round to slightly ovoid in shape. They generally contain a single round nucleus eccentrically set in a small abundant amount of dark blue cytoplasm that occasionally contains a few pink granules. The nuclei exhibit a coarsely granular chromatin pattern. Moderate anisocytosis is noted. Hematoxylin and eosin; ×60 magnification; bar = 20 μm.

Two years following initial presentation, the patient was re-presented to the neurology service for follow-up examination. Neurologic status at this time was normal, however owners noted a circular cutaneous mass located at the tail base. Current medications were prednisolone (0.8 mg/kg PO q 24 h) and melphalan (0.1 mg/kg PO q 72 h). Diagnostics performed included CBC and chemistry panel, follow-up cervicothoracic MRI, FNA of tail base mass, protein electrophoresis, and Bence-Jones protein test. Complete blood count abnormalities were persistent leukopenia (3.0 K/μl, reference range: 6.0–18.0 K/μl) and thrombocytopenia (134 K/μl, reference range: 3.0–12.0 K/μl). Serum panel abnormalities included persistent hyperglobulinemia (5.0 g/dl, reference range: 2.3–3.8 g/dl). Magnetic resonance imaging of the cervicothoracic spine revealed resolution of previous abnormalities (Figure 2). Fine needle aspiration of the tail base mass was consistent with plasma cell tumor (Figure 3). Protein electrophoresis revealed a narrow peak in the gamma range (Gamma 1, 1.81 g/dl, reference range: 0.3–2.5 g/dl). Although within the reference range, the shape and overall protein profile was strongly suggestive of monoclonal gammopathy. Bence Jones proteins were negative. Given the definitive diagnosis of cutaneous plasma cell tumor, the patient was again referred to our oncology service to address disease progression. Melphalan was temporarily discontinued due to moderate leukopenia. At consultation with the oncology service 2 weeks later, repeat CBC revealed resolving leukopenia. Cyclophosphamide in lieu of melphalan was offered but declined. The owners elected to reinitiate melphalan due to known benefits indicated by a normal neurologic exam and resolution of the T2 vertebral lesion. Over the following 8 months, the tail base mass enlarged, and additional cutaneous masses developed, one of which was confirmed via histopathologic evaluation as plasma cell tumor. Repeat staging was offered but declined by the owner. Several chemotherapies: cyclophosphamide, chlorambucil, and lomustine were trialed but unsuccessful at halting disease progression. Three years following initial diagnosis, the patient remained neurologically normal, but owners requested elective euthanasia due to progression of cutaneous masses.

## Discussion

In cats, SOP is less frequent than in humans or dogs. Much of the information used to diagnose, treat, and assess treatment response is inferred from limited human data. In people, diagnosis requires advanced imaging confirming a single lesion, histopathological confirmed plasmacytoma, bone marrow biopsy confirming no or insignificant bone marrow plasmacytosis, and immunohistochemical findings consistent with monoclonality. In this case report, clinical signs, MRI, bone marrow FNA, and clinicopathologic findings were used to diagnose SOP. Additionally, MRI was used to assess treatment response, which documented complete resolution of the SOP.

In humans, SOP has a median age of onset of 55 years, younger than that of MM, with a slight male predilection (12, 14, 17). Clinical signs are associated with neuroanatomic localization. Like humans, veterinary SOP patients tend to be younger than MM patients, however no breed or sex predilections exist. Perhaps this is due to minimal published veterinary data. Like published literature (2, 5–8), the patient in this case report initially presented with nebulous clinical signs and hyperglobulinemia noted on bloodwork. Fourteen days of disease progression caused neurologic signs consistent with a neuroanatomic localization of C6-T2 with the lesion later identified at the T2 vertebra. Of significance to the presumptive SOP diagnosis, the patient had persistent hyperglobulinemia later classified as a monoclonal gammopathy and proteinuria. In retrospective studies of MRD in cats (2, 7), hyperglobulinemia and proteinuria were the most common laboratory findings. These clinical findings are far less common in humans with SOP (14) and not required for a diagnosis. Recent published guidelines (13, 14) suggest that diagnosis of SOP should consist of bone marrow aspiration and biopsy with <10% monoclonal plasma cells. Morphology and degree of plasma cell infiltration should be assessed by flow cytometry or immunohistochemistry given the prognostic significance. At the time of this case report, flow cytometry was not the recommended guideline and not used. However, the patient did have bone marrow analysis performed. The authors acknowledge that the core sample was not diagnostic, but the FNA did not reveal plasmacytosis. Additionally, monoclonality, a diagnostic criterion in humans, was present.

Human guidelines recommend CT or MRI for diagnosis of SOP. Additionally, full-body fluorine-18-fludeoxyglucose positron emission tomography/computed tomography (18F-FDG PET/CT), an imaging technique used to identify hyper-metabolic bone lesions, has shown prognostic value recently (13). Current recommendations are that it be combined with CT or MRI as both may offer complimentary information. At the time of presentation of this case, 18F-FDG PET/CT was not the recommended guideline nor routinely used in veterinary medicine due to availability and cost. MR imaging performed in this case report showed homogenous reduction in T1 and T2 weighted signal intensity of the entire T2 vertebra relative to all other vertebral bodies. The caudal aspect of the T2 vertebra and ventrally located soft tissue was homogenously contrast enhancing. These MRI findings, while not pathognomonic for SOP are consistent (18–20). Other primary bone tumors, like osteosarcoma, chondrosarcoma, and fibrosarcoma tend to have aggressive imaging findings and were deemed less likely. Other round cell neoplasia (e.g., lymphoma, multiple myeloma), infectious granuloma or metastatic disease were differential diagnoses. The authors acknowledge that advanced imaging of the entire spine was not performed. However, neuroanatomic localization was not suggestive of multifocal disease and full spinal radiographs were performed by the primary veterinarian with no significant abnormalities noted. Histopathologic confirmation of a SOP was not obtained. However, infectious diseases reported to cause a monoclonal gammopathy (21, 22) were excluded. And unlike humans, MRD in cats tend to present with liver and splenic involvement (2). In this case, ultrasound and fine needle aspirate of both organs was not consistent with plasmacytosis. Presence of significant plasmacytosis (<5%) was not present in bone marrow fine needle aspirates. Together, all diagnostic results did not show evidence of an infectious agent nor myeloma elsewhere. A presumptive diagnosis of SOP was made.

Treatment options for feline solitary osseous plasmacytoma include palliative, chemotherapy, and radiotherapy. Surgery may be recommended if vertebral instability is noted but is not a mainstay of therapy. Radiotherapy is considered the gold-standard treatment in humans (12–14), but limited information is available in veterinary medicine. A recent retrospective canine study by Elliott et al. (23) evaluated the response and outcome to plasma cell tumors and found it to be a useful therapeutic modality with tumor response often complete and prolonged. However, response to therapy was subjectively measured by externally palpable tumor volume and when available, imaging. Specifically, post-radiotherapy radiographs were available for 2/4 SOP patients and neither showed reduction of disease despite improvement in clinical signs. In this case study, radiotherapy was offered but declined due to cost. Extrapolating from human medicine (12–14) and a single feline SOP case report (9), melphalan chemotherapy and prednisolone was instituted. Clinical response was seen within 3 days of initiating therapy and maintained for 2 years. Complete resolution of the T2 vertebral lesion was noted on 2-year follow-up via MRI. Use of advanced imaging to confirm disease resolution or document disease progression is a recommendation in human medicine (12–14). The patient in this case did develop histopathological confirmed cutaneous plasmacytoma at a new location which does suggest disease progression, a reported phenomenon of SOP. Complete re-staging of this patient was not performed excluding myeloma elsewhere in the body however, resolution of the previously documented solitary lesion was confirmed. This is the first case report describing the use of MRI to diagnose and document complete resolution of this neoplasm in the feline patient following melphalan. The case study confirms that combination melphalan and prednisolone therapy can resolve SOP, offering prolonged disease-free interval and good quality of life. The authors recommend that it be offered when radiotherapy is not an option.

## Data Availability Statement

The original contributions presented in the study are included in the article/supplementary material, further inquiries can be directed to the corresponding authors.

## Author Contributions

TM, ST, and RB contributed to management of the case. HH was responsible for the magnetic resonance imaging. CT was responsible for the clinicopathological evaluation. TM wrote the original draft of the manuscript. All authors participated in the revision and approval of the final manuscript.

## Funding

Open access publication fees was supported by University of Tennessee College of Veterinary Medicine.

## Conflict of Interest

The authors declare that the research was conducted in the absence of any commercial or financial relationships that could be construed as a potential conflict of interest.

## Publisher's Note

All claims expressed in this article are solely those of the authors and do not necessarily represent those of their affiliated organizations, or those of the publisher, the editors and the reviewers. Any product that may be evaluated in this article, or claim that may be made by its manufacturer, is not guaranteed or endorsed by the publisher.
